# Embedding mental rehearsal in surgery: a comprehensive review of the evidence

**DOI:** 10.3389/fsurg.2025.1524468

**Published:** 2025-06-13

**Authors:** Josephine Walshaw, Bright Huo, Paul Barach, Philippa Banks, Adam McClean, Florent Lebon, Faisal Mushtaq, David Jayne, Danilo Miskovic, Marina Yiasemidou

**Affiliations:** ^1^ Leeds Institute of Medical Research, St James’s University Hospital, University of Leeds, Leeds, United Kingdom; ^2^ Medical School, Dalhousie University, Halifax, NS, Canada; ^3^ Thomas Jefferson University, Philadelphia, PA, United States; ^4^ Faculty of Medicine, Imperial College London, London, United Kingdom; ^5^ Sheps Health Services, Research Center, University of North Carolina, Chapel Hill, NC, United States; ^6^ Department of General Surgery, Bradford Teaching Hospitals NHS Foundation Trust, Bradford, United Kingdom; ^7^ Department of Psychology, Université de Bourgogne, Dijon, France; ^8^ School of Psychology, University of Leeds, Leeds, United Kingdom; ^9^ St Mark’s Hospital, London North West University Healthcare NHS Trust, London, United Kingdom; ^10^ The Royal London Hospital, Barts Health NHS Trust, London, United Kingdom; ^11^ Department of Health Research, University of York, York, United Kingdom

**Keywords:** education, surgery, simulation, mental rehearsal (MR), training

## Abstract

**Background:**

Mental rehearsal (MR), the deliberate practice of skills specific to a procedure, has been successfully used in sports and music training for decades, but has not been adopted in surgery. This narrative review explores MR's role in surgical training and clinical practice, evaluating its effectiveness in motor skill acquisition, technical and non-technical skill development, and real world clinical implementation. Our aim was to assess MR's impact on both surgical education and clinical performance, while identifying the barriers to its routine adoption in surgical training.

**Methods:**

We searched for relevant studies on the topic and impacts of MR in surgery using the Medline database up to December 2024. A range of studies were included covering mental rehearsal, surgical education, surgical training, and surgical outcomes. The primary outcomes were to provide insights into the mechanisms and implementation of MR in surgery and to assess the potential impact of MR on surgical outcomes.

**Results:**

The narrative review provides scientific insights into the mechanisms of MR in surgery and describes in detail the implementation methodology. The majority of evidence demonstrates that MR is beneficial when used as an adjunct approach to other forms of training. Moreover, there is evidence to support MR as a low-cost and valuable learning technique. Many questions remain regarding training schedules including the optimal duration and nature of the MR sessions, accommodating the surgeon's prior experience, optimal number of repetitions, and addressing the abilities of the participants to perform mental imagery. Most studies have heterogenous methods, diffuse aims and poor descriptions of the specific intervention components. Several studies applied MR in demanding real-life surgical environments and demonstrated feasibility in surgery.

**Conclusions:**

The preliminary findings suggest that MR may improve the performance of operators and operating teams as an efficient adjuvant to traditional surgical skills training methods. More work is needed to better understand how MR interventions can best be implemented to improve training, practice, and outcomes in routine surgical practice.

## Introduction

Mental rehearsal (MR) is a cognitive training technique that encompasses both mental and motor imagery (1–3). Mental imagery broadly refers to the simulation of experiences, while motor imagery refers to imagined actions. Neuroscientific evidence suggests that imagined movements engage the same neural networks as executed actions (4), with kinaesthetic imagery adhering to the constraints of human sensorimotor control (5–8). This indicates that MR may produce a similar learning effect to “hands-on” practice through neurocognitive mechanisms. MR offers the opportunity to practice specific procedures or related skills before performing complex tasks on patients, potentially impacting patient outcomes (9).

The two main modalities used in MR are visual and kinaesthetic imagery. Visual imagery involves the mental visualisation of action without explicit physical movement (1–3), whereas the kinaesthetic imagery involves the mental reproduction of movement and actions. Mental images can be formulated in different ways; from direct perceptual information, stored long-term memory, or a combination of both, and can be either generic or specific depending on the context, participants and goals (10). MR may be used by novices and expert operators to advance procedure learning and expertise (11).

Despite growing interest in MR within surgical training, there remain several gaps in the literature. There is no consensus on how best to implement MR in a structured training program, the optimal duration and frequency of sessions, or its long-term impact on surgical performance. Moreover, there is a lack of practical guidance for educators on how to practically integrate MR into surgical curricula. This comprehensive narrative review aims to (i) provide an overview of MR as a motor skill learning method, (ii) evaluate its effectiveness in improving task performance, as measured by assessments of the technical and non-technical skills of the operators and operating teams, and (iii) offer practical recommendations for incorporating MR in surgical training programmes.

## Methods

This study is a narrative review aimed at synthesising existing literature on mental rehearsal (MR) in surgical education. The review included five key phases: Identifying the research question, identifying relevant studies, study selection, collating data, and synthesising results.

A structured search was conducted on Medline to identify relevant articles published up to December 2024. The search terms included “*mental rehearsal*,” “*mental imagery*,” “*surgical training*,” and “*simulation.*” Additional studies were identified through manual searches of reference lists in key articles.

We included studies if they examined the role of MR in surgical training, assessed its impact on technical and non-technical skill acquisition, or provided insights into its implementation. Opinion pieces, conference abstracts, and studies focusing on non-surgical applications of MR were excluded. Given the narrative review design, no formal quality assessment or meta-analysis was conducted. Instead, findings were synthesised thematically to provide an overview of MR's effectiveness and practical applications in surgical education.

### Mental rehearsal and motor skill acquisition

Cognitive and motor processes share common features, including an improved performance with practice and a decline with a lack of it. In the initial stages of both imagery practice and motor skills learning, the objectives are identical: to build a mental representation of the task. Feedback plays an essential role in both, leading to automation with repeated practice (4).

The application of MR in surgical training is supported by established theories of motor learning and skill acquisition. Schmidt's schema (12), a classic motor learning theory, emphasises the importance of mentally storing how a task looks, feels, and sounds. This mental storage modifies the feedback processes that are fundamental components of visual and kinaesthetic MR. This suggests that MR may strengthen motor schemas by reinforcing mental representations of key procedural steps. Identifying the learner's mental models—understanding how novices perceive a procedure as compared to experts—has been a common method for preparing MR scripts to train novices (13, 14).

Additionally, Fitts and Posner's Three-Stage Model of Skill Acquisition (15) describes learning in three phases: the cognitive stage (understanding the task), the associative stage (refining movement), and the autonomous stage (performing with minimal cognitive effort). MR aligns particularly well with the cognitive and associative stages, where mental imagery can facilitate the transition from explicit learning to procedural execution.

Experimental evidence indicates that the time required to perform a task mentally is proportional to the time needed for physical performance (16). Visual perception and memory also play a significant role in the quality of mental imagery, affecting the vividness and accuracy of the rehearsed actions (17–20). Motor imagery is also subject to similar computational models as overt motor actions, including alterations in performance caused by sensory feedback and other factors regulating movement response (5, 21–23).

Common cortical and subcortical networks observed by neuroimaging studies demonstrate that MR engages motor-related networks, including the supplementary motor area (24, 25), parietal cortex (26–29), premotor cortex (25, 26, 30), primary motor area (27, 31, 32) and cerebellum (33). Moreover, electroencephalography (EEG) studies demonstrate a substantial overlap in the electrical activity between real and imagined actions (34–36). A source analysis from EEG studies also point to the primary motor structures being implicated in both mental and physical task performance (36). Similarly, potential current signals appear both quantitatively and qualitatively equivalent for imaginary and actual task performance (34, 37).

Beyond motor learning, MR may also optimise cognitive load management and decision-making during surgical tasks. Cognitive Load Theory (38) suggests that MR can reduce extraneous cognitive demands, allowing trainees to allocate more working memory resources to task execution. Furthermore, the Stress Inoculation Model (39) posits that MR under simulated stress conditions may enhance psychological resilience and decision-making under pressure, a crucial factor in high-risk surgical environments (40). These cognitive theories provide additional support for MR's integration into surgical training beyond its effects on motor performance alone. Despite these insights, there is currently no standardised theoretical model guiding MR protocol development or implementation in surgery, leading to variability or lack of its routine use in training approaches.

### Preparation, content, and duration of mental rehearsal sessions

The most popular approaches for designing an MR session for surgery are (i) creating a consensus between experts on how the task should be performed (13, 14, 41), (ii) preparing physician/educator-led cognitive task analysis sessions which include a breakdown of the procedure steps in order to facilitate the visualisation process (42–46), or (iii) a combination of the two (47). A consensus is achieved through a series of semi-structured interviews and consequent thematic analysis of their transcripts in search of visual and kinaesthetic cues. This process yields a script that requires subsequent validation (13, 14, 41).

The structure and duration of MR sessions in surgery varies greatly in most studies. Mulla et al. (45) provided medical students with a 25-min one-on-one mental training session with clinical instructors, which included step-by-step descriptions of the motor skills required, relaxation techniques, as well as intrinsic and extrinsic visualisation of the chosen assessment tasks. The students were asked to undergo 15-min daily self-driven practice sessions. In contrast, three RCTs provided a single 30-min session to participants which consisted of psychologist-led relaxation techniques, step-by-step breakdowns of procedures embedded with sensory cues and surgeon-supervised mental imagery rehearsal sessions. Of these, two showed a favourable effect of MR on surgical skills (43, 44) and one study showed no difference (42). Two additional RCTs employed several 30-min MR sessions immediately prior to the surgical procedure. These were based on a MR script that was preceded by relaxation techniques. Both studies showed superior performance in the MR-trained group (13, 14).

Currently, the duration of MR prior to operating is not standardised. An RCT by Cragg et al. (48) spanning four months initially provided a 3-h training session on MR, followed by a practice schedule coupled with a multi-sensory self-evaluation form. At the end of the 2nd month, a 30-min telephone discussion was conducted with further MR advice from a consultant. After the 3rd month participants were instructed to develop a “script” of procedural steps, and after four months a summative 20-min independent session was undertaken before their final assessment. Kaulfuss et al. (49) performed an RCT with medical students who completed a formal training program, received a demonstration by a laparoscopic expert, began 30 min of hands-on training, followed by 60 min of formal theoretical training, and underwent a supervised mental training session prior to two weeks of independent MR. Participants who engaged in MR performed better and faster, and remarkably the skills improvement was noted to last several months. Immenroth et al. (50) offered the longest duration of one-on-one mental training sessions, lasting 90 min. In contrast, some studies planned sessions lasting merely 5 min (51) and 3 min (47) with participants asked to repeat the process several times prior to their assessments.

A systematic review of RCTs by Goble et al. (52) found that in studies where MR groups showed improved outcomes, the median duration of each session was 30 min and the training lasted for a median of 24 days. Notably, it is unclear whether there is a linear relationship between MR duration and surgical performance. Indeed, prolonged motor imagery sessions may have adverse effects on motor performance, due to mental fatigue (53). Alongside delineating an optimal time, the pragmatics of conducting MR in a busy hospital setting with staffing shortages and productivity pressures also need to be considered.

In anticipation of the MR sessions, non-experts must become familiar with the tasks taught (13, 14, 43, 45, 51). Various training techniques have been proposed for this purpose. Eldred-Evans et al. (44) followed Peyton's four-step teaching approach while others trained novices to proficiency before applying the MR intervention (14) or employed expert teaching (42, 43). Once the non-expert surgeon gains a basic understanding of the task, they practise several times during the supervised session(s). At the end of this process, the surgeon may be assessed to ensure their understanding of the task. Equally important, they receive guidance on how to mentally practise, before engaging in a series of supervised or self-driven MR sessions (13, 14, 43–45, 51).

An evaluation may be completed to establish a surgeon's baseline ability to practise mentally, as the capability to mentally reconstruct images varies widely across individuals. A systematic review by Suica et al. (54) identified imagery ability evaluation methods and evaluated their psychometric properties. The best-rated assessments were the Movement Imagery Questionnaire (MIQ) and its iterations (MIQ-R and MIQ-3), and the Vividness of Movement Imagery Questionnaire-2 (VMIQ-2) for evaluation of motor imagery ability. Regarding mental imagery evaluation, only the Sport Imagery Ability Questionnaire (SIAQ) and VVIQ showed sufficient psychometric properties.

### Outcome measures to assess mental rehearsal impact

Validated outcome measures are needed to provide meaningful feedback to the learner, in the form of a composite score, an interpretation of results or a risk categorisation of the surgical task. No consensus exists about the most effective outcome measures in the study of MR (55). The most frequently used measure in studies is the overall performance metric. Multiple RCTs have utilised this technique with significant heterogeneity of assessed factors, including a composite of any number of the following: time taken to complete a task, accuracy (44, 45, 47) and rating checklists (13, 14, 41–43, 50, 51). The Objective Structured Assessment of Technical Skill (OSATS) is a validated tool for the assessment of surgical skills (56) and has been implemented in multiple studies assessing MR (13, 50).

An indirect way to assess the MR sessions is through questionnaires (13, 14, 41) addressed to supervising experts or the participating trainees. This method assesses the performance characteristics, and mental characteristics including stress, teamwork, and confidence. Arora et al. (13) applied the validated State-Trait Anxiety-Inventory (STAI) to assess the impact of MR training on the stress levels of novice surgeons completing virtual laparoscopic cholecystectomies. The outcome measures in the literature are frequently limited to short-term performance metrics, with little regard paid to the confounding and potential biasing factors as well as the long-term benefits (55). A lack of consistent outcome measures has limited the use of meta-analysis in assessing the effectiveness of MR in surgery, and larger trials with consistent and validated outcome measures are required (57).

### Mental rehearsal for surgical skill acquisition

MR was demonstrated to be effective in teaching both basic technical skills and full procedural tasks across various surgical disciplines. Table 1 provides an overview summary of key studies on MR in surgical training. Studies assessing fundamental surgical skills have demonstrated MR's effectiveness in improving tasks such as suturing and precision cutting. For example, previous studies have used MR to train surgeons in cutting a 44 mm diameter circle from a rubber glove using a box trainer or a virtual reality (VR) simulator (44, 45), opening and closing a midline incision of an anaesthetised rabbit (42, 43), handsewn intestinal anastomosis on bovine intestine (58), or suturing during a laparoscopic Nissen fundoplication on a VR simulator (47). Similarly, MR has been applied to full procedural tasks, including a simulated laparoscopic cholecystectomy on a VR simulator (13, 14), a porcine liver and gallbladder placed in a box trainer (50), laparoscopic jejunojejunostomy (41), and a cricothyroidectomy on a mannequin (51). The diversity in difficulty and subspecialty of the chosen tasks demonstrates that MR can be an effective tool for all training grades and across all surgical specialties.

**Table 1:** Summary of key studies on mental rehearsal (MR) in surgical training, stratified by study setting.

Study	Sample size	Procedure	Intervention(s)	MR length	Outcome measures	Key findings
Educational/non-clinical setting						
Sanders et al. (42)	65 s year medical students	Basic surgical skills	(1) 3 sessions of physical practice on suturing (2) 2 sessions of physical practice and 1 session of MR (3) 1 session of physical practice and 2 sessions of MR	All training sessions were 30 min, and carried out at 1 week intervals	7 item global rating scale: incision, needle handling, suturing, flow, quality of sutures, respect for tissue, knowledge of procedures	Physical practice followed by MR was statistically equal to additional physical practice
Bathalon et al. (51)	44 first year medical students	Cricothyrotomy (simulation)	(1) MR and KG (2) KG alone (3) Standard advanced trauma life support approach	1 h teaching session	OSCE testing the performance of the different steps, time, and fluidity	MR with KG improved the overall total score KG alone or with MR improved fluidity
Immenroth et al. (50)	98 surgeons	Laparoscopic cholecystectomy (simulation)	(1) Additional MR training (2) Additional practical training (3) No additional training	One-to-one mental training lasting 90 min with additional 30 min alone	OSATS instrument and the global rating scale	Performance at follow-up was significantly better in the MR and control groups but not the practical training group
Sanders et al. (43)	64 s year medical students	Incision and suturing a pig's foot. Incision and suturing live rabbit.	Usual skills course (didactic lectures, demonstrations, physical practice) plus either			
(1) MR training (2) textbook study	2 sessions of guided relaxation and MR session lasting approximately 30 min	Rating scales, surgical skills checklist, experience questionnaire, degree of self-confidence toward surgery, trait anxiety, state anxiety, and the revised minnesota paper form board test	Significant interaction favouring the MR group over the textbook study group. Measures of attitude toward surgical skill and anxiety as a personality trait were not significantly related to performance			
Arora et al. (13)	20 novice surgeons	Laparoscopic cholecystectomy (VR)	VR curriculum, followed by either			
(1) MR (2) unrelated activity (control)	30 min of MR using a validated protocol before each VR laparoscopic cholecystectomy	Subjective stress levels (STAI), objective stress (continuous heart rate, salivary cortisol), validated mental imagery questionnaire	MR group showed significantly lower subjective and objective stress. Improved imagery was associated with lower stress.			
Arora et al. (14)	20 novice surgeons	Laparoscopic cholecystectomy (VR)	VR curriculum, followed by either			
(1) MR (2) unrelated activity (control)	30 min of MR using a validated protocol before each VR laparoscopic cholecystectomy	OSATS global rating scale, validated mental imagery questionnaire	MR group had significantly superior performance in all 5 cholecystectomy sessions. Improved imagery was associated with higher quality of surgical performance.			
Jungmann et al. (47)	40 medical students	Laparoscopic knot tying in a Nissen Fundoplication model	Two sessions of laparoscopic basic training on VR simulator. The intervention group completed MR between training sessions	Informed how to practice, handout (demonstration video and checklist of task), advised to practice on at least 4 different days between sessions	Time, tip trajectory	Additional MR did not improve the overall knot-tying performance
Wetzel et al. (69)	16 surgical residents (minimum 2 years residency and experience in vascular surgery)	Carotid endarterectomy (crisis simulation)	Intervention between two simulations			
(1) Stress management training (SMT): Coping strategies, MR, relaxation (2) No intervention	MR performed as part of the SMT package, length not stated	Realism of simulation scenario, stress levels (STAI, observer rating, heart rate, salivary cortisol), surgical performance (OSATS, OTAS, EPA, decision making), how useful the SMT was on surgical competence	SMT showed enhanced OTAS, increased coping skills, and reduced stress. Qualitative analysis revealed improved technical skills, decision making, and confidence. MR and the booklet were rated as most useful			
Mulla et al. (45)	41 medical students	Basic laparoscopic skills (cut out a circle)	(1) No training (2) Box training (3) Box training with additional practice session (4) VR simulator training (5) MR	One-to-one 30-min MR training, 3 sessions of 15-min MR at home over 3 days	Time, precision, accuracy, performance	MR group ranked last in all domains
Eldred-Evans et al. (44)	64 medical students without laparoscopic experience	Basic laparoscopic skills (cut out a circle)	(1) No additional training (2) Additional VR training (3) Additional MR training (4) VR and MR training with no box training	Conducted by experienced mental trainer, length not stated	Time, accuracy, precision, overall performance	MR group demonstrated improved laparoscopic skills in VR and box assessment
Louridas et al. (41)	20 senior surgical trainees	Porcine laparoscopic jejunojejunostomy part of roux-en-y gastric bypass (as part of a crisis scenario)	(1) Conventional training (2) MR group	In-person instruction from psychologist. Relaxation exercise, MR guided by scripts, provided with videos and voice-over of script. 7 days to practice independently	OSATS, bariatric OSATS (BOSATS), objective and subjective stress parameters, non-technical skills rating tool	Improvement in OSATS and BOSATS scores with MR. No difference in stress levels or non-technical skills
Yiasemidou et al. (67)	20 junior surgical trainees (PGY 2–4)	Laparoscopic cholecystectomy (VR)	(1) Didactic video (control) (2) MR with 3D visual aids (3) MR only	25-min MR session (one group with interactive 3D visual aids, one without)	Performance (Instrumental tip path length, number of movements, time to extract gallbladder), safety metrics (number of non-cauterised bleeding, number of perforations, damage to vital structures)	Control group took longer to complete procedure. MR with 3D aid made few movements. No difference for safety metrics.
De Witte et al. (70)	28 resident surgeons (PGY 1)	Laparoscopic suturing and knot tying	(1) Basic surgical skills + additional cognitive training (motor imagery and action observation) (2) Basic surgical skills only (3) No additional training	2 sessions per day for 2 days (8-min video of expert laparoscopic suturing followed by 8-min MR session)	Surgical performance (OSATS), spatial abilities (mental rotation test, spatial orientation test), mental workload (NASA task load index)	MR did not enhance laparoscopic suturing performance in a short, intensive course. High cognitive load may have prevented skill acquisition.
Cragg et al. (48)	21 general surgical trainees (PGY 3–5), complete data available for 14 participants	Laparoscopic cholecystectomy (porcine model)	(1) Surgical cognitive simulation (2) Control group	MR performed for 20 min before procedures—included MR, goal setting, thought management	Objective performance measures (16 domains from ISCP PBA, global performance score, time to complete), subjective measures (confidence, decision making, focus, preparedness, stress, fatigue, learning ability)	Cognitive stimulation reduced procedure time and improved global performance score. Improved self-reported preparedness, focus, confidence, stress, decision-making, teamwork
Kaulfuss et al. (49)	24 medical students with no prior laparoscopic experience	Two laparoscopic exercises (peg transfer, cutting a circle)	(1) Control group (read and analysed urology paper) (2) Video training group (3) Video and MR group	Structured MR training session, instructed to practice independently for 2 weeks	Performance (global rating scale, binomial checklist, procedure time), cognitive and psychological measures	MR improved long-term surgical performance (significantly higher scores at 16 month follow up)
Souiki et al. (58)	17 first semester surgical residents and interns	Hand-sewn intestinal anastomosis performed on bovine intestine	(1) MR group (relaxation, guided MR, individual MR) (2) Control group (reviewed written protocol)	Total of 45 min MR training—5 min relaxation session, 10 min guided MR, 30 min individual MR	Surgical performance score	Mean overall score was significantly higher in the MR group
Modi et al. (71)	12 surgical residents	Laparoscopic knot-tying	(1) MR group (2) Textbook reading group	Five repetitions of a 90-second guided audio script	Functional near-infrared spectroscopy (prefrontal and motor cortical activation), technical performance (leak volume, objective performance score, task progression score)	MR reduced prefrontal cognitive demands, improving motor cortical efficiency, and leading to better technical performance
Clinical/intraoperative setting						
Komesu et al. (66)	68 surgical residents who had observed at least 1 cystoscopy and performed ≤3 cystoscopies	Cystoscopy	(1) MR of cystoscopy (2) Reading a standard text chapter describing cystoscopy	One-to-one MR session lasting <20 min, within 24–48 h of each cystoscopy	GSOP, helpfulness of preparation using visual analog scale	MR surgical assessment scores were significantly higher. No difference in operative times between groups. MR rated more helpful than control.
Patel et al. (79)	15 combined procedures (9 pre-intervention, 6 post-intervention)	Combined open/endovascular arterial procedures	A structured MR performed at the commencement of the endovascular phase	All members of the theatre team paused to perform 5 min of MR	Error rates, average delay, average danger	MR significantly lowered error rates, delay and danger scores
Yiasemidou et al. (60)	49 patients, consultant surgeons (number not stated)	Low anterior resection	(1) Routine practice (2) MR using MRI scans (3) MR using virtual 3D models (4) MR using printed 3D models	MR session lasted 20–30 min, performed within 48 h before the operation	Surgical performance (CAT, OCHRA), length of stay, complications (clavien-dindo), specimen quality	MR did not affect CAT or OCHRA scores. Time for dissection was shorted for MR with MRI scans compared to routine practice

KG, kinesiology; OSCE, objective structured clinical examination; VR, virtual reality; GSOP, global scale of operative performance; STAI, state-trait-anxiety-inventory; OTAS, observational teamwork assessment for surgery; EPA, end product assessment; PGY, post-graduate year; CAT, competency assessment tool; OCHRA, objective clinical human reliability analysis; PBA, procedure based assessment.

MR can be applied across all levels of competency, from novice learners developing basic technical skills to experienced surgeons refining advanced procedures. For example, junior trainees can rehearse core surgical steps, such as achieving access to the abdominal cavity, while more experienced trainees can practise procedural sequences they have seen but not yet performed (59). Experienced surgeons can utilise MR to rehearse variations that could potentially occur during technically demanding procedures (4, 60). Skervin et al. (61) demonstrated that 91.5% of consultants and surgical trainees incorporate MR informally prior to operating, suggesting that this approach is likely to be widely accepted by trainees and consultants. Interestingly, a 2017 systematic review found that ten out of fourteen trials demonstrated objective improvement in surgical skills, with more obvious benefits to mature surgeons with greater levels of expertise (62). This is in keeping with a review by Anton et al. (63) who highlighted that MR is most useful when applied to surgeons of different experience levels as a supplemental training tool to refine and improve their surgical skills.

Multiple systematic reviews support the efficacy of MR in surgical training. Rao et al. (57) conducted a systematic review of nine randomised controlled trials (RCTs) involving 474 participants, with five trials reporting significant improvement in skill acquisition. These studies examined both basic tasks, such as precision cutting, and full procedures, including laparoscopic cholecystectomy. Assessment tools varied, incorporating objective measures (e.g., checklists, time to complete task, number of instrumental tip movements) alongside non-objective measures. Interestingly, the studies that found neutral outcomes tended to rely on verbal instruction or brief video demonstrations, that featured short MR sessions (≤30 min), and required unsupervised self-rehearsal periods, suggesting that structured, repeated MR sessions may yield better outcomes.

Gabbott et al. (64) conducted a systematic review of eight studies with 268 participants, evaluating MR's impact on teamwork and non-technical skills. While five studies demonstrated improvements in technical performance, only three studies reported a positive impact on teamwork performance. However, the heterogeneity across different scales, outcomes, and outcome measures weakens the inferences from this study. Similarly, Snelgrove and Gabbott (65), reviewed six studies and found that two-thirds of the primary literature supported MR's role in improving both technical and non-technical skills, including surgical movements, communication, and teamwork.

A systematic review by Rajarathnam et al. (55) found that 13 of 19 studies demonstrated improved performance when MR was combined with traditional training. Multiple RCTs have shown that MR is superior to textbook learning in both instructor-assessed and self-assessed technical skills (43, 66). Additionally, Yiasemidou et al. (67) found that MR was superior to video-based learning in improving laparoscopic cholecystectomy skills. Another review looking specifically at MR in orthopaedic surgery included 11 studies and found significant benefits, with cognitive training associated with notably improved surgical performance and increased knowledge when compared with traditional methods of learning, such as textbooks, slide shows, and videos (68).

It should be noted that while there is some evidence to suggest that MR in combination with practical training is beneficial over practical training alone (44), its use as an alternative may be detrimental, with one RCT showing lower operative speed and precision in the MR-trained group as compared to both box trainers and VR simulation (45). However, the study's impact was limited due to methodological limitations and heterogeneity, as well as the lack of large sample sizes and long-term learning assessments.

Arora et al. (13) performed an RCT assessing the stress levels of novice surgeons during simulated laparoscopic cholecystectomies. They found that the group who had undergone prior MR training had significantly lower heart rates, cortisol levels, and self-assessed stress levels as compared to the control group. Wetzel et al. (69) demonstrated similar results, while additionally identifying increased teamwork in the MR group. However, De Witte et al. (70) demonstrated an increase in cognitive load in MR study participants leading to detrimental learning outcomes when compared with those trained with traditional teaching methods alone.

A recent study by Modi et al. (71) used functional near-infrared spectroscopy (fNIRS) to assess the impact of MR on prefrontal and motor cortical activation during a laparoscopic knot-tying task. The authors demonstrated that mental rehearsal (MR) can enhance surgical skill acquisition by reducing prefrontal cognitive load, increasing motor cortical activation, and improving technical performance. The MR participants had better suture quality (lower leak volume, *p* = 0.019) and higher objective performance scores (*p* = 0.043) as compared to a textbook reading control group. These findings support the neurocognitive benefits of MR in optimising surgical task execution.

### Mental rehearsal compared against simulation training

MR sessions can be conducted in various settings, either independently or as an adjunct to simulation-based training. Simulation is a well-established tool in surgical education and is widely recognised as an effective adjunct to traditional training (72). However, a frequent criticism of the current simulation approaches in surgery is the inability to fully replicate the experience of conducting a surgical operation and the limited ability to show lasting behaviour change outcomes (73). While simulation actively engages trainees with physical models, VR systems, or cadaveric specimens, mental rehearsal (MR) relies solely on cognitive imagery, allowing surgeons to visualise procedural steps, anticipate challenges, and engage sensory cues without physical execution of movements. Some hybrid models integrate MR with simulation, but these should be distinguished from pure MR techniques.

Unlike simulation, MR is not limited to visual reconstruction alone, but also engages auditory (74), olfactory (75), and haptic (76) imagery, potentially providing a more holistic representation of the real-world theatre environment. This cognitive flexibility makes MR particularly valuable for preparing surgeons to manage anatomical variations and intraoperative decision-making. For example, during a laparoscopic cholecystectomy, MR can be used to mentally reconstruct the dissection of Calot's triangle, allowing the surgeon to anticipate anatomical variations (e.g., proximal bifurcation of the cystic artery), and the potential associated complications (e.g., common bile duct injury).

Another key advantage of MR is its accessibility and cost-effectiveness. Simulation often requires expensive equipment, dedicated facilities, and instructor time, making widespread adoption challenging (72, 77). In contrast, MR has minimally associated costs and can be performed anytime, anywhere, making it universally accessible (55). While supervised MR sessions may enhance learning, they can also be effectively practised independently after initial training (13, 14). However, it is worth noting that unsupervised simulation training has been shown to yield less behaviour modification and performance improvement as compared to structured guidance (78), a factor that should be explored further in MR research.

MR has broad applications in surgical training. Experienced surgeons can use it to refine techniques for complex procedures (60). For others, it may be a cost-effective way of maintaining basic technical skills whilst on a career break or for clinician scientists who divide their time between research and surgery. Additionally, simulation and likely MR are useful for shortening the surgeon's learning curve for an operation (4), thus reinforcing patient safety by allowing surgeons to rehearse critical steps before performing them in real world settings. Given its flexibility, MR represents a valuable complement to simulation-based training, providing an alternative when physical simulation is unavailable and enhancing cognitive preparedness for surgery.

### Implementation in a real-world surgical environment

The integration of MR in a real-world clinical setting is challenged by time limitations and the lack of consultant supervision given the growing demand for service provision. Studies that have implemented MR in actual surgical environments (as opposed to simulation-based training) suggest that MR can be performed pre-operatively to enhance technical preparedness, decision-making, and efficiency, without causing delays in surgical care and productivity (60, 66, 79). However, the majority of MR research remains focused on educational and training settings, with fewer studies evaluating its impact on live surgical performance.

Yiasemidou et al*.* (60) compared MR combined with virtual and physical patient-specific anatomical models and Magnetic Resonance Imaging (MRI) to routine preparation prior to surgery. The study recruited expert surgeons, who performed MR prior to commencing minimally invasive low anterior resections; the MR session focused on the pelvic dissection part of the procedure only. The primary outcome was the quality of the surgery. The study demonstrated no differences between the groups, and undertaking MR caused no delays in surgery. The only significant difference the authors found was the reduction in idle time during surgery for the group that performed MR using pre-operative MRIs as visual modalities.

Patel et al. (79) assessed error incidence before and after implementing a structured MR prior to the endovascular phase of combined open/endovascular arterial procedures. A trained observer assessed 15 combined procedures over a period of six weeks. Two blinded assessors carefully examined surgical event logs for technical errors. When they identified errors, these were categorised based on type, potential to harm the patient (danger), and potential to disrupt the procedure (delay). After conducting nine procedures, a focus group introduced a structured MR before the endovascular phase for the subsequent six combined procedures. The goal was to improve the surgical process and potentially reduce surgical errors and prevent patient harm. The error patterns were compared before and after the implementation of the MR technique to measure the impact of the MR intervention. After the application of MR, the error rates during the endovascular phase significantly decreased as compared to the rates before the intervention. Additionally, both the danger and delay scores were reduced (1.2 errors per dangerous event and 1.3 errors per delayed event, respectively) in comparison to the scores before the MR. The authors concluded that the MR may reduce the frequency and severity of surgical errors and patient harm.

Komesu et al. (66) assessed whether MR prior to cystoscopy can improve surgical performance. The authors conducted an RCT involving multiple centres. Residents with ≤3 previous cystoscopies were randomly assigned to two different preparation methods before the surgery: one group received preoperative mental imagery sessions, while the other group read a book chapter that described a cystoscopy. The primary focus of the study was to compare the surgical performance scores between the two groups. Additionally, they also measured the operative times and collected feedback from surgical residents about how they perceived the helpfulness of their preparation methods. A total of 68 residents participated in the randomisation process, with 33 assigned to the imagery group and 35 to the control group. The two groups were similar in terms of age, cystoscopic procedure experience, residency year level, and gender distribution. The residents who underwent mental imagery sessions before the surgery achieved surgical assessment scores that were 15.9% higher compared to the control group, with the difference statistically significant (*p* = 0.03). Furthermore, the residents who received the mental imagery preparation rated it as significantly more helpful as compared to the control group. However, there were no significant differences in the operative times between the two groups.

A qualitative study by Jolly et al. (80) explored ten Australian general surgery trainees' perceptions of MR using semi-structured interviews. All trainees reported using MR before operations, but this was informal and unstructured. The study highlighted that MR could address key challenges in surgical education, including reducing anxiety, improving communication, and enhancing training during periods of reduced clinical exposure. Participants supported the formal integration of MR into surgical curricula, with a preference for online training resources to mitigate time constraints. Standardisation of training was seen as a major benefit, although concerns were raised about variability in surgical techniques. The study concluded that mental training has the potential to supplement existing surgical education and should be implemented through a structured, accessible format to optimise trainee preparedness.

## Discussion

Our comprehensive review provides a practical guide and support for the effective integration of MR in surgical training and the ongoing maintenance of surgical competency. We propose a structured, stepwise approach to facilitate its adoption, outlining key steps for implementation (Table 2), and ensuring that MR is tailored to learners' and training program needs while complementing existing training modalities. We highlight several successful strategies across different experience levels, assessed through various learning and clinical outcomes. A critical question remains: how can MR be effectively implemented in real-world surgical environments without disrupting patient care or busy clinical workflows?

**Table 2:** A stepwise framework for implementing mental rehearsal (MR) in surgical training.

Step	Implementation strategy	Key considerations
1. Define learning objectives	Identify specific technical and non-technical skills to enhance through MR	Tailor objectives based on trainee level and surgical complexity
2. Select MR modality	Choose between visual imagery (mental visualisation) and kinaesthetic imagery (rehearsing movements)	Combine both modalities for a comprehensive approach
3. Develop MR script	Use cognitive task analysis to break procedures into structured, step-by-step mental exercises	Incorporate sensory cues (e.g., tactile, auditory) for realism
4. Determine training schedule	Establish session duration, frequency, and integration with simulation or live training	Short, frequent sessions (e.g., 10–15 min) may be more effective than long, infrequent ones
5. Facilitate instructor support	Provide guided MR sessions with expert feedback and peer discussion	Encourage self-directed MR after initial instructor-led training
6. Assess performance and adapt	Use validated tools (e.g., OSATS) to evaluate MR effectiveness and modify sessions accordingly	Gather feedback from trainees to refine program design

We found that MR is a more accessible and potentially more cost-effective technique than existing educational training such as simulation and reading of scientific materials. These findings raise a series of questions about why MR has not been routinely embedded in surgical curricula. What are effective guidelines for how best to embark on embedding MR in training contexts? What curricula design models are most effective from a pedagogical perspective? We highlight the merits of MR, while attempting to make the case to the surgical community on the need for the surgical community to seriously support implementing MR as an effective, inexpensive surgical competency training approach.

A key distinction in the MR literature is its application in controlled training environments vs. real-world surgical settings. In training settings, MR is primarily used as a cognitive and motor skill acquisition tool, helping trainees develop technical proficiency, procedural understanding, and confidence before hands-on practice. Studies conducted in simulation-based settings have reported improvements in task execution speed, error reduction, and stress management following MR interventions. However, the transition from training to real-world implementation is poorly defined, and the use of MR in surgical environments remains limited and inconsistent.

Real-world studies have focused on assessing MR's impact on intraoperative workflow, decision-making efficiency, and patient safety. These studies suggest that preoperative MR may enhance intraoperative efficiency, reduce idle time, and lower surgical error rates. However, due to limited sample sizes and variability in study design, it remains unclear whether MR consistently translates into improved postoperative outcomes and patient safety. There is currently no standardised protocol for implementing MR in surgical settings, and further research is required to evaluate its feasibility, scalability, and long-term impact on surgical outcomes and hospital workflow.

Unlike learning-focused studies, implementation research studies have primarily been descriptive rather than analytical, making it difficult to assess the true feasibility and long-term impact of MR in surgical practice. There are several variables that require calibration before MR is widely endorsed for effective implementation. For instance, the success of MR is highly associated with the expertise of the participants (81–83), therefore the duration and nature of the MR sessions may vary according to the surgeon's experience, support and competency levels. The MR sessions for non-experts should include a familiarisation stage with the procedure or task, followed by an assessment to ensure understanding. Expert surgeons can omit this step and instead focus on full sensory engagement and continuous repetition in a personally chosen environment to achieve optimal results. MR has been shown to increase movement accuracy (84, 85) and quality (86), however, the number of repetitions necessary for learning surgical procedures is unknown and needs to be evaluated through robust research. The variance in baseline ability to perform MR should also be addressed. Could the discrepancy in MR outcome results be due to the diverse MR baseline ability of the participants? Assessing baseline ability through validated questionnaires and through focus groups, as well as ethnographic observations to help determine the optimal MR method (visual, haptic, or other) for each surgeon. A personalised, targeted approach will enhance surgeon engagement, MR outcomes and improve their skill acquisition.

### Limitations

It is important to acknowledge the limitations associated with our review. First, the evidence base on MR remains heterogeneous, with significant variability in study designs, outcome measures, and intervention protocols, making direct comparisons challenging. Most studies have focused on short-term performance metrics, with few evaluating long-term skill retention or clinical outcomes. Second, there is a lack of standardized pedagogical models guiding MR implementation, leading to inconsistencies in training approaches across studies. Third, from a methodological perspective, this review was narrative rather than systematic, meaning that the search strategy, study selection, and data synthesis were not conducted using formal systematic review methodology. While the narrative review approach allows for a broad and flexible synthesis of MR applications, it does not provide a meta-analysis or formal quality appraisal of included studies. Finally, many of the studies done on MR impact assume fixed external team and hospital environments, and yet other unmeasured changes such as staffing and workflow changes might have impacted the studies putative benefits.

### Future directions

Future research should seek to integrate validated skill acquisition models and surgical performance theories to improve the design, consistency, and reproducibility of MR interventions. The current variability in MR protocols, training duration, and outcome measures highlights the need for a standardised framework that can be applied across different surgical specialties and levels of expertise. In addition, more robust studies are needed to determine the long-term impacts of MR on surgical performance and patient outcomes, particularly in real-world settings. While existing evidence suggests MR can enhance technical proficiency and decision-making, few studies have assessed its sustained effects on skill retention, intraoperative efficiency, and postoperative complications.

Prospective, multicentre trials and longitudinal studies are needed to explore how MR influences surgeon competencies over time, how it integrates with existing training methods, and whether its benefits translate into improved patient care. Furthermore, research is needed to focus on addressing the logistical and practical challenges of implementing MR in high-pressure surgical environments, ensuring that it can be seamlessly incorporated into routine practice without disrupting workflow or increasing cognitive load. Future studies can help establish MR as a reliable, evidence-based training tool that can optimise both learning outcomes and clinical performance in surgery.

## Conclusions

Our review explored the neurocognitive mechanisms underlying mental rehearsal (MR), its current applications in surgical education, and its potential future role in optimising surgical performance and patient outcomes. MR offers promising benefits for surgeon training by enhancing both technical and non-technical skills, improving cognitive preparedness, and may serve as a cost-effective adjunct to traditional training methods. More work is needed to bridge the gaps between theory and practice, requiring a continuous loop of knowledge generation, dissemination, and uptake on how best to provide surgical training under real world conditions. Our paper provides a training framework for integrating MR more effectively into surgical training. Future research is needed on standardising training protocols, novel study designs that improve statistical and logistical efficiency, and evaluating long-term patient and learner outcomes to maximise MR effectiveness in surgical education.
